# Establishment of local norms and standardization of the MATRICS consensus cognitive battery in Chinese adolescents

**DOI:** 10.3389/fpsyt.2025.1675079

**Published:** 2025-09-17

**Authors:** Yupan Tan, Xiaofen Zong, Hongjie Li, Mengyao Feng, Jinxin He, Xia Sun, Yuanyuan Zhang, Xin Wang, Jie Yao, Maolin Hu

**Affiliations:** ^1^ Department of Psychiatry, Renmin Hospital of Wuhan University, Wuhan, Hubei, China; ^2^ Department of Psychiatry, Xiaogan Mental Health Center, Xiaogan, Hubei, China

**Keywords:** MCCB, adolescents, norms, schizophrenia, Chinese

## Abstract

**Background:**

The MATRICS Consensus Cognitive Battery (MCCB) is a widely used, standardized tool for assessing cognitive impairments in schizophrenia clinical trials. However, normative data for the MCCB in healthy adolescents remain scarce. This study establishes the first regionally representative norms for the MCCB in Chinese adolescents aged 11-16 years.

**Methods:**

Using stratified cluster sampling, we recruited 1,244 participants from Xiaogan City, demographically matched to regional population statistics. The Chinese-adapted MCCB assessed seven cognitive domains through nine subtests. Comprehensive analyses evaluated age, gender, and education effects using ANCOVA and non-parametric tests for non-normal distributions.

**Results:**

Our analyses revealed distinct developmental patterns across cognitive domains. Significant age-related improvements emerged in most measures (all p<0.05 except Trail Making Test-Part A), demonstrating continuous rather than stage-like progression. Gender differences were observed, with males outperforming in cognitive domains of processing speed, reasoning and problem solving, working memory and attention/vigilance, while females showed advantages in visual learning. After adjusting for gender, age initially exhibited significant associations with all cognitive tests; however, upon further adjustment for education, the effect of age was no longer significant in eight of the nine tests.

**Conclusion:**

This study establishes the first locally representative normative standards for the MCCB in Chinese adolescents, addressing a crucial gap in culturally sensitive cognitive assessment tools. Our findings demonstrate the complex interplay of age, gender, and educational factors in cognitive development, providing clinically valuable benchmarks for early detection of schizophrenia in this population.

## Introduction

Neurocognitive dysfunction represents a core feature of schizophrenia (1). To develop standardized methods for evaluating novel treatments aimed at mitigating these cognitive deficits, the National Institute of Mental Health (NIMH) launched the Measurement and Treatment Research to Improve Cognition in Schizophrenia (MATRICS) initiative. This endeavor culminated in the creation of the MATRICS Consensus Cognitive Battery (MCCB), a reliable and valid assessment tool for clinical trials (2, 3), which is highly sensitive to detecting cognitive dysfunction in schizophrenia (4). Although MCCB co-norms have been established for healthy adults (3, 5, 6) and adults with schizophrenia (7), norms for adolescent populations have not yet been established, particularly given that cognitive impairment often emerges during the premorbid phase of schizophrenia, which typically occurs during adolescence, and persists throughout the lifespan (8).

The MCCB comprises nine core tests that systematically evaluate six dimensions of neurocognitive impairment in schizophrenia: attention/vigilance, speed of processing, working memory, visual learning, reasoning and problem solving, and verbal learning (9). Building on emerging research highlighting the importance of social cognition in real-world functioning, a tenth test, i.e., the Managing Emotions component of the Mayer-Salovey-Caruso Emotional Intelligence Test (MSCEIT) (2), was integrated to assess the social cognition domain. Validation studies demonstrate the MCCB’s clinical utility. Performance improvements on the MCCB correlate with real-world functional gains (10, 11). Additionally, its psychometric properties have been validated through the MATRICS Psychometric and Standardization Study (7) and the Validation of Intermediate Measures studies (12). Its 44 language versions facilitate global use, though normative data vary cross-culturally. For example, Singaporean English speakers performed differently than Americans due to cultural/educational factors (13), mirroring findings in other populations (14). Such variations underscore the need for culturally-specific norms, motivating China normative data collection.

A significant proportion of individuals with schizophrenia experience illness onset or prodromal symptoms during adolescence (15–17), and evidence shows adolescents with schizophrenia-spectrum disorders exhibit consistent deficits across MCCB domains except Social Cognition compared to healthy peers (18, 19). Thus, establishing normative MCCB trajectories for healthy adolescents is critical. Current limitations in adolescent normative data hinder clinical interpretation, necessitating studies to delineate developmental patterns across age groups, genders, and education levels. Although Norwegian (20) and North American (21) studies have begun to delineate MCCB developmental trajectories in adolescents, comprehensive, directly applicable normative data for this critical age group remain scarce internationally. The absence of adolescent normative data substantially limits clinical interpretation of MCCB results, particularly considering the high incidence of schizophrenia onset and prodromal symptoms during adolescence (8).

Addressing these methodological limitations, this study establishes regionally representative normative standards for the MCCB in Chinese adolescents. Utilizing a stratified cluster sampling methodology, we identified Xiaogan City in Hubei Province as our study site - a demographically representative midsize city randomly selected from Hubei’s prefecture-level municipalities. Our sampling framework is designed with reference to China’s education system, focusing on covering three distinct educational stages: primary school (high grade), junior high school, and senior high school. From the collected samples, it randomly selects adolescents aged 11-16 (grade 5-12) in proportion to the age distribution specified in the XiaoGan Statistical Yearbook (2024). This approach captures both essential neurodevelopmental milestones and regionally specific sociodemographic characteristics. The resulting normative data will provide clinically valuable reference standards for psychiatric assessment in Central China while simultaneously establishing foundational metrics for future cross-cultural investigations of neurocognitive development.

## Methods

### Participants and recruitment process

Participants were adolescents aged 11 to 16 years from Xiaogan City, Hubei Province. Stratified random sampling was conducted by educational stage, based on the official school list provided by the Xiaogan Education Bureau, with 2 schools randomly selected from each stage. In each selected schools, 2 classes were randomly selected from each target grade (grades 5-12). Prior to the survey, written informed consent was obtained from all students in these classes and their guardians, with explicit notification that participants could withdraw at any time if experiencing discomfort (without adverse consequences). No withdrawals occurred, so all students in the selected classes completed the assessment. According to the XiaoGan Statistical Yearbook (2024), the sample was selected with corresponding age and gender ratios. Six age groups (11–16 years) were selected with proportional distribution: 11-year-olds (15.35%), 12-year-olds (16.24%), 13-year-olds (17.60%), 14-year-olds (18.65%), 15-year-olds (16.64%), and 16-year-olds (15.51%). The sample comprised 51.77% male and 48.23% female participants. In accordance with China’s education system, participants were categorized by educational attainment: 4 years (5.23%), 5 years (12.94%), 6 years (13.59%), 7 years (23.15%), 8 years (16.72%), 9 years (8.92%), 10 years (16.40%), and 11 years (3.05%).

Inclusion criteria: aged 11–16 years at recruitment; native Mandarin speakers with the ability to complete interviews and questionnaires in Chinese; no known chronic somatic diseases affecting brain function.

Exclusion criteria: history of past or present diagnosis of major psychiatric disorders; participants with first-degree relatives known or suspected to have major psychiatric disorders; active or recent (within the past month) substance abuse; neurological/medical comorbidities; intelligence quotient (IQ) < 70; or significant sensory/communication impairments.

All participants and guardians provided written informed consent. The study was approved by the Medical Ethics Committee of Renmin Hospital of Wuhan University (Approval No. WDRY2024-K039).

### Neuropsychological measures

To evaluate cognitive functioning, the Chinese adaptation of the MATRICS Consensus Cognitive Battery (MCCB) (22, 23) was administered. This instrument measures six neurocognitive dimensions: Speed of processing [Category fluency/verbal fluency for animals (VF), Symbol coding subtest from the Brief Assessment of Cognition in Schizophrenia (SC-BACS), Trail Making Test-Part A (TMT-A); Attention/vigilance (Continuous Performance Test-Identical Pairs, CPT-IP); Working memory (Spatial Span subtest from the Wechsler Memory Scale-III, WMS-III SS); Verbal learning (Hopkins Verbal Learning Test-Revised, HVLT-R); Visual learning (Brief Visuospatial Memory Test-Revised, BVMT-R); Reasoning and problem solving (Mazes subtest from the Neuropsychological Assessment Battery, NAB Mazes); and one Social Cognition domain (Managing Emotions component of the Mayer–Salovey–Caruso Emotional Intelligence Test, MSCEIT).

The MCCB comprises 10 core tests across seven cognitive domains, nine of which were administered in this study. Due to cultural adaptation challenges as there is no corresponding alphabet system in Chinese, the Letter-Number Span (LNS) test was not adopted.

### Data analysis

The distribution normality of all nine test scores was evaluated using the Lilliefors test (24), and significantly skewed distributions (p < 0.05) were subjected to transformation. Among square, logarithmic, and Box–Cox transformations, the most suitable method was selected to ensure the post-transformation p-value exceeded 0.05 and the distribution approximated normality; original scores were retained if transformation was ineffective. Raw scores of the nine MCCB subtests were converted to T-scores using the formula: T-score= 10 + 3× (raw score - Mean)/Standard Deviation. For the TMT-A, which exhibits an opposite trend of scores compared to other subtests, a specialized standardization method was employed, with the specific formula as follows: T-score = 10 - 3 × (raw score - Mean)/Standard Deviation. This transformation was based on the raw score distribution of our sample (n = 1,244). For each subtest, we first calculated the mean and standard deviation (SD) of raw scores across the entire sample. Using the formula above, T-scores were standardized such that the mean T-score for each subtest was 10 and the SD was 3. Subsequent differential analysis explored age, gender, and educational effects on nine subtest T-scores. Analysis of covariance (ANCOVA) assessed performance disparities related to age and educational attainment, while independent T-tests evaluated gender effects. For non-normally distributed data, rank sum tests (Mann-Whitney U or Kruskal-Wallis) were applied to assess group differences. To clarify the relative roles of age and years of education in performance across 10 cognitive domains, we conducted hierarchical multiple linear regression analyses. The scores of each domain served as dependent variables, with gender included as a covariate. Predictors were incorporated in three sequential steps: Model 1 included only gender; Model 2 added age to Model 1; and Model 3 further added years of education to Model 2. Standardized regression coefficients (β) were used to compare the effect magnitudes of age and education. Changes in model explanatory power (ΔR²) and significance tests were employed to evaluate the unique contribution of education relative to age.

## Results

### Demographic characteristics

The sample had a mean age of 13.52 years (SD = 1.66) and an average educational attainment of 7.42 years (SD = 1.88). The cohort comprised 600 females (48.23%) and 644 males (51.77%), reflecting a balanced gender distribution.

### Age effects

**Figure 1:**
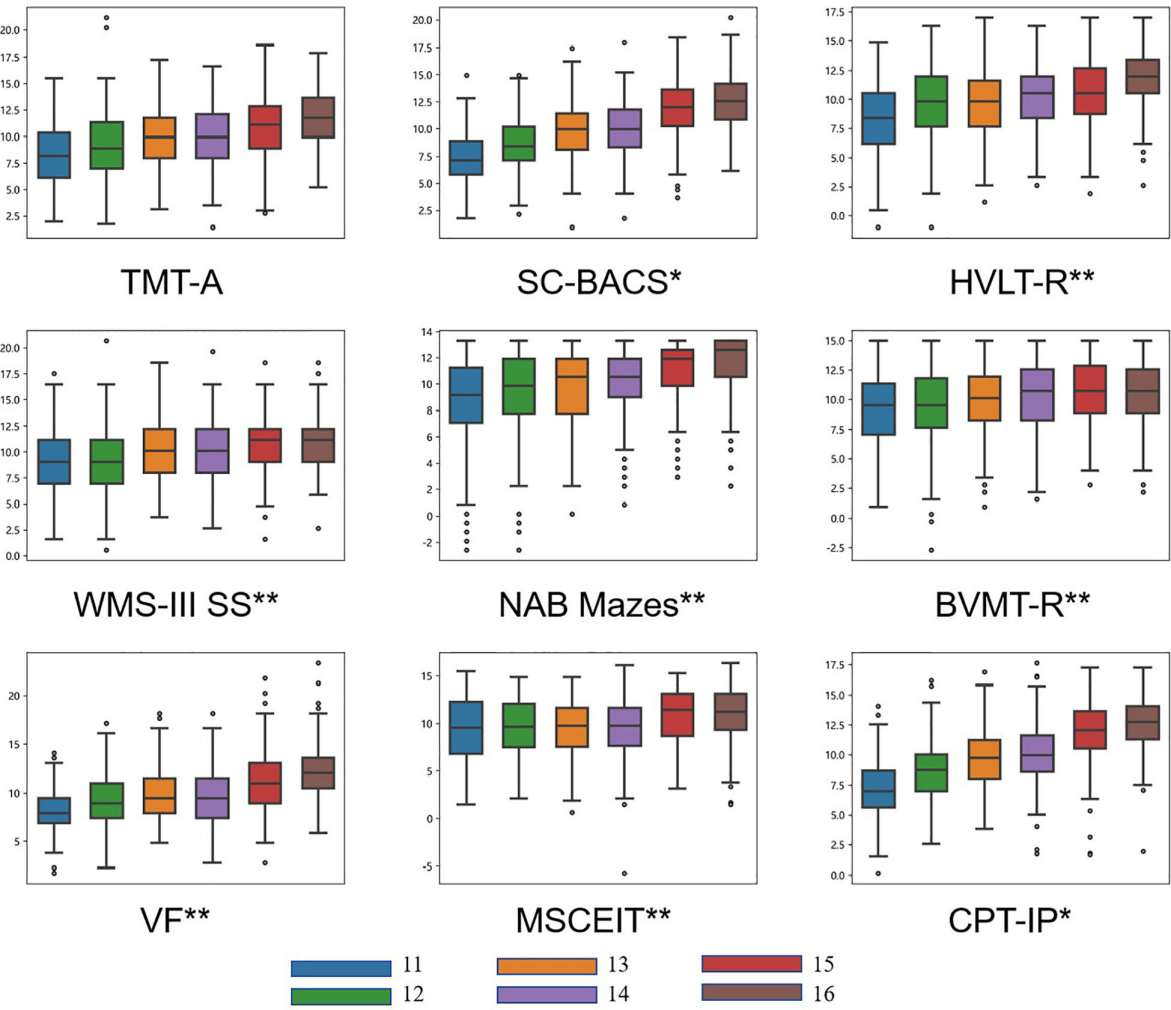
Mean T-scores of the MCCB by age group. The figure presents nine box plots showing the distribution of scores for the cognitive tests from the MCCB: TMT-A, SC-BACS, HVLT-R, WMS-III SS, NAB Mazes, BVMT-R, VF, MSCEIT and CPT-IP. Each test is represented by a separate box plot, with data points stratified into six age groups (11 to 16 years old), each denoted by a distinct color. Overall between-group differences are indicated by asterisks (* for p < 0.05; ** p < 0.001).

Given the differences in educational attainments across the six age groups, education was treated as a covariate in ANCOVA models examining age-related effects. Non-parametric Kruskal–Wallis tests were employed for non-normally distributed data. Significant age effects were observed in the tests of BACS (F = 2.73, p =0.018), HVLT (H = 132.10, p < 0.001), WMS (H = 73.78, p < 0.001), NAB Mazes (H = 123.59, p < 0.001), BVMT (H = 52.06, p < 0.001), VF (H = 223.40, p < 0.001), MSCEIT (H = 58.55, p < 0.001), and CPT-IP (F = 2.23, p =0.049), except for the TMT-A test (F = 1.13, p =0.342) (see **Table 1** and **Figure 1**).

**Table 1:** Mean T-scores for the 9 MCCB tests, by age group.

AGE	11years (n=191)	12years (n=202)	13years (n=219)	14years (n=232)	15years (n=207)	16years (n=193)	F	p
TMT-A	8.24 ± 2.67	9.10 ± 3.02	9.95 ± 2.60	9.99 ± 2.82	10.94 ± 2.99	11.76 ± 2.61	1.13	0.3423
SC-BACS	7.25 ± 2.39	8.55 ± 2.42	9.79 ± 2.36	10.04 ± 2.49	11.83 ± 2.56	12.47 ± 2.48	2.73	0.018
CPT-IP	7.16 ± 2.32	8.61 ± 2.37	9.71 ± 2.50	10.09 ± 2.46	11.82 ± 2.65	12.54 ± 2.22	2.23	0.049
							H	p
HVLT-R	8.40 ± 3.16	9.49 ± 2.96	9.61 ± 2.99	10.11 ± 2.61	10.73 ± 2.81	11.64 ± 2.48	132.10	<0.001
WMS-III SS	8.75 ± 3.14	9.29 ± 2.93	10.28 ± 2.86	9.95 ± 3.01	10.87 ± 2.74	10.78 ± 2.76	73.78	<0.001
NAB Mazes	8.66 ± 3.49	9.26 ± 3.09	9.77 ± 2.88	10.13 ± 2.71	10.8 ± 2.64	11.35 ± 2.35	123.59	<0.001
BVMT-R	9.07 ± 3.13	9.28 ± 3.19	10.05 ± 2.94	10.15 ± 3.01	10.84 ± 2.53	10.54 ± 2.8	52.06	<0.001
VF	8.18 ± 2.3	9.12 ± 2.68	9.81 ± 2.55	9.61 ± 2.85	11.19 ± 3.01	12.13 ± 2.86	223.40	<0.001
MSCEIT	9.42 ± 3.34	9.62 ± 2.94	9.58 ± 2.86	9.57 ± 2.96	10.9 ± 2.71	11.00 ± 2.77	58.55	<0.001

ANCOVA, education as covariance; Kruskal-Wallis test.

A consistent age effect was observed across all cognitive tests except TMT-A: later adolescent consistently outperformed younger ones, and this effect remained significant after controlling for education. Scores peaked at age 16 and showed an overall monotonic increase with age, though BVMT and WMS scores plateaued after peaking at age 15.

### Gender effects

**Figure 2:**
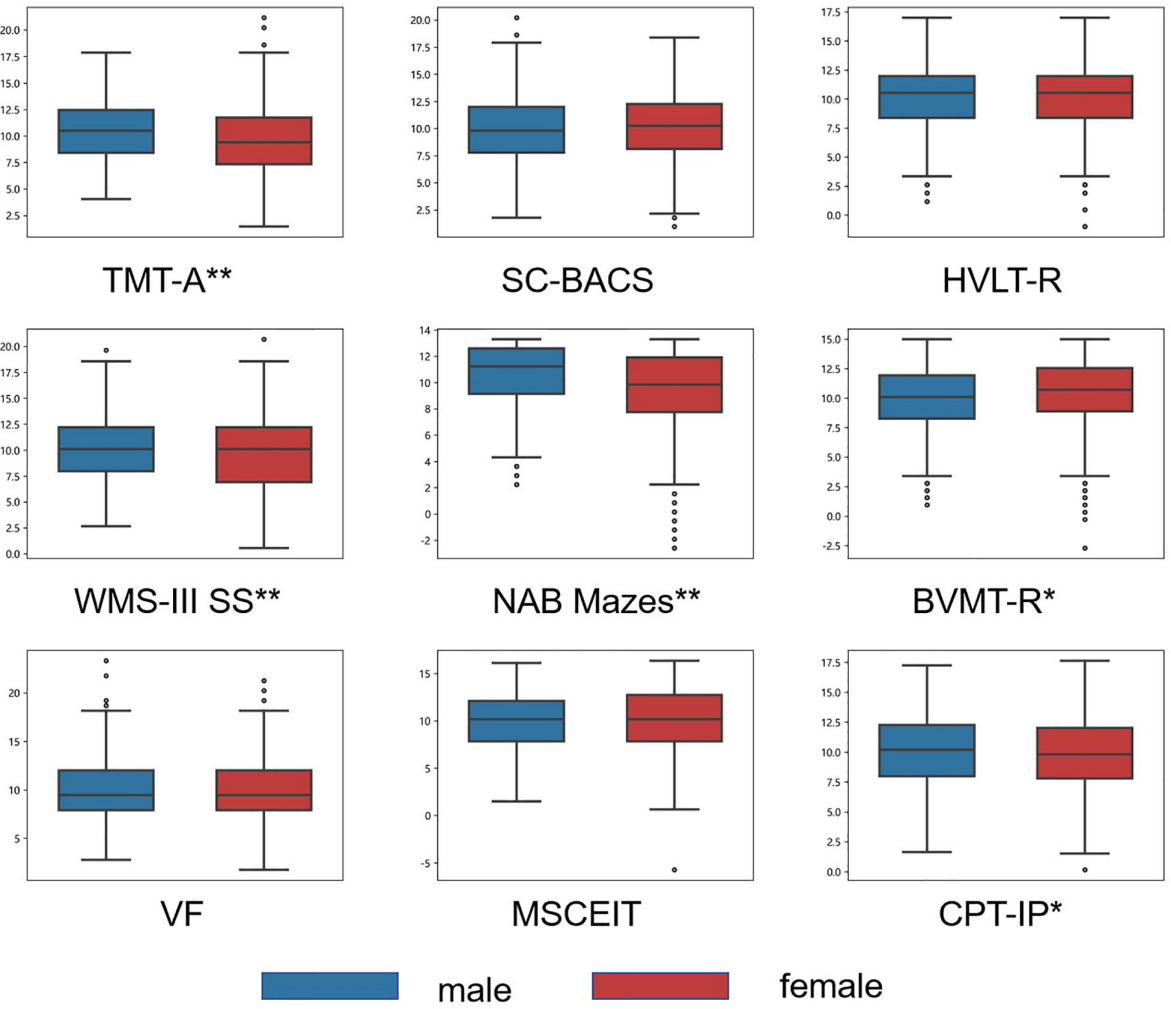
Mean T-scores of the MCCB by gender group. Box plots display the distribution of scores for males (blue) and females (red) on nine cognitive tests from the MCCB. Significant between-sex differences for each test are indicated by asterisks (* p < 0.05; ** p < 0.001).

Significant gender differences were observed on several cognitive tests. In the TMT-A (t = 5.49, p < 0.001), CPT-IP (t = 2.12, df = 1242, p =0.033), WMS (U = 216913.5, p < 0.001), and NAB Mazes (U = 244081, p < 0.001), males performed better than females. Conversely, females outperformed males in BVMT (U = 178759, p =0.022). There were no statistically significant differences between male and female in the other cognitive tests (see **Table 2** and **Figure 2**).

**Table 2:** Mean T-scores for the nine MCCB tests, by gender.

Gender	Male (n=644)	Female (n=600)	t	p	Effect size
TMT-A	10.45 ± 2.75	9.52 ± 3.18	5.49	<0.001	0.31
SC-BACS	9.84 ± 3.09	10.17 ± 2.89	-1.92	0.055	-0.11
CPT-IP	10.17 ± 3.06	9.81 ± 2.93	2.12	0.033	0.12
			U	p	
HVLT-R	10.04 ± 2.88	9.96 ± 3.12	192783.5	0.948	
WMS-III SS	10.33 ± 2.86	9.65 ± 3.11	216913.5	<0.001	
NAB Mazes	10.70 ± 2.47	9.25 ± 3.32	244081	<0.001	
BVMT-R	9.86 ± 2.93	10.15 ± 3.07	178759	0.022	
VF	10.01 ± 2.98	9.99 ± 3.03	194163	0.879	
MSCEIT	9.96 ± 2.89	10.04 ± 3.11	188835	0.491	

t-test for normal data, Mann-Whitney U test.

### Education effects

**Figure 3:**
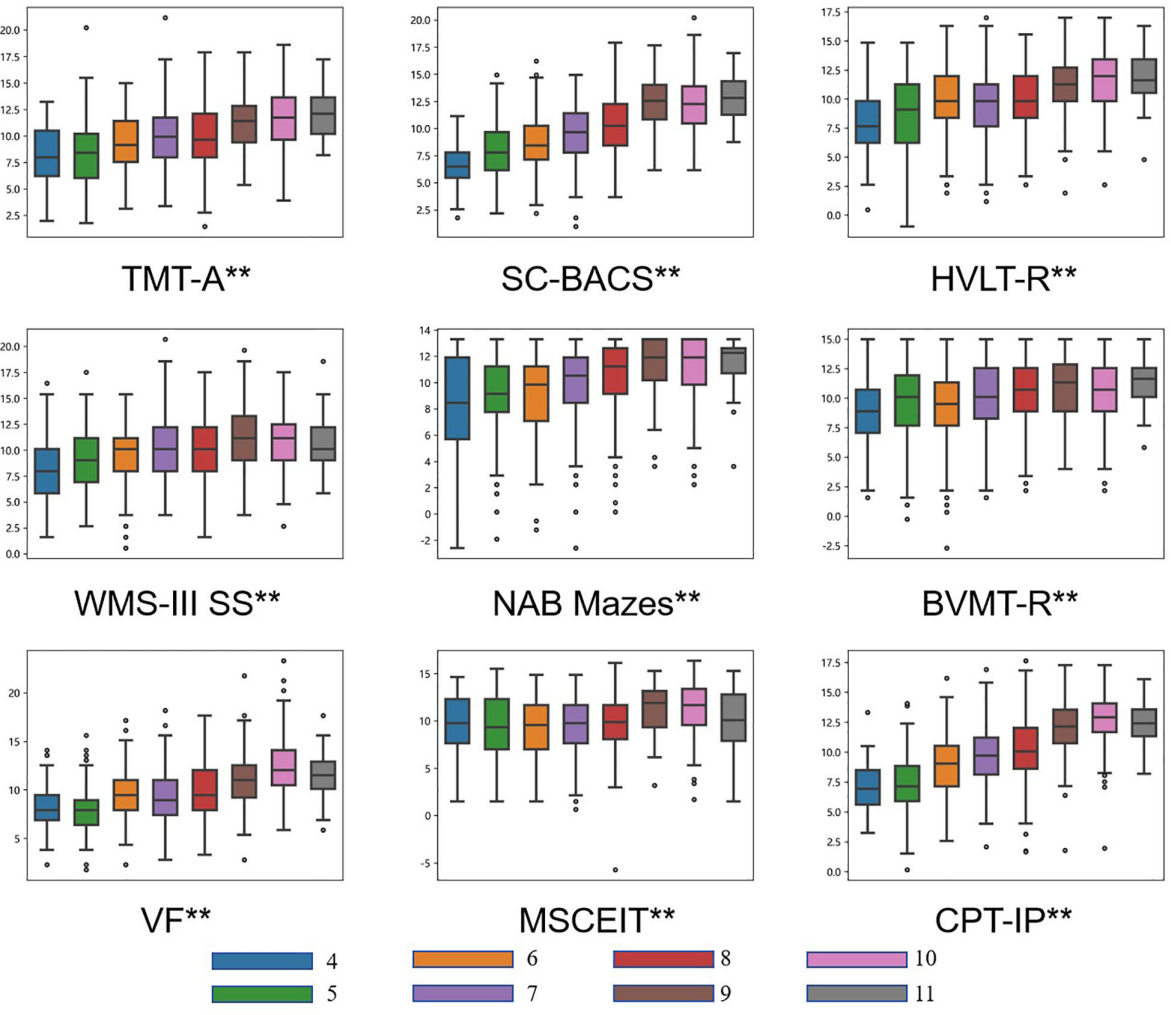
Mean T-scores of the MCCB by education level. Nine box plots display the distribution of scores for the nine cognitive tests from the MCCB. Each test is stratified by education level, categorized into groups from 4 to 11 years of schooling and represented by distinct colors. Significant overall between-group differences are indicated by asterisks (** p < 0.001).

To account for age-related variance in cognitive performance, we included age as a covariate in our analysis of education effects. Educational attainment significantly affected performance on all nine cognitive tests of TMT-A (F = 3.54, p < 0.001), BACS (F = 9.05, p < 0.001), HVLT (H = 161.76, p < 0.001), WMS (H = 77.51, p < 0.001), NAB Mazes (H = 134.02, p < 0.001), BVMT (H = 53.78, p < 0.001), VF (H = 254.87, p < 0.001), and MSCEIT (H = 76.74, p < 0.001)(see **Table 3** and **Figure 3**).

**Table 3:** Mean T-scores for the 9 MCCB tests, by education group.

Education	4 (n=65)	5 (n=161)	6 (n=169)	7 (n=288)	8 (n=208)	9 (n=111)	10 (n=204)	11 (n=38)	F	p
TMT-A	8.09 ± 2.74	8.35 ± 2.93	9.22 ± 2.66	10.02 ± 2.71	9.82 ± 2.89	11.29 ± 2.58	11.64 ± 2.91	11.92 ± 2.33	3.54	<0.001
SC-BACS	6.64 ± 2.14	7.81 ± 2.46	8.63 ± 2.51	9.61 ± 2.34	10.25 ± 2.55	12.47 ± 2.14	12.34 ± 2.55	12.93 ± 1.89	9.05	<0.001
CPT-IP	7.02 ± 2.08	7.31 ± 2.37	8.92 ± 2.44	9.62 ± 2.42	10.24 ± 2.67	11.94 ± 2.45	12.74 ± 2.11	12.41 ± 1.86	9.47	<0.001
									H	p
HVLT-R	7.67 ± 2.91	8.73 ± 3.23	9.84 ± 2.88	9.66 ± 2.96	9.81 ± 2.54	11.12 ± 2.87	11.62 ± 2.41	11.76 ± 2.29	161.76	<0.001
WMS-III SS	8.15 ± 3.31	9.02 ± 2.88	9.49 ± 2.87	10.01 ± 3.01	10.21 ± 2.93	11.16 ± 2.79	10.86 ± 2.75	10.26 ± 2.69	77.51	<0.001
NAB Mazes	8.12 ± 4.21	8.93 ± 2.99	9.22 ± 2.89	9.82 ± 2.95	10.29 ± 2.75	11.26 ± 2.27	11.08 ± 2.62	11.49 ± 2.03	134.02	<0.001
BVMT-R	8.77 ± 3.16	9.48 ± 3.1	9.1 ± 3.26	9.99 ± 2.97	10.27 ± 2.95	10.87 ± 2.44	10.58 ± 2.79	11.22 ± 2.04	53.78	<0.001
VF	8.43 ± 2.41	8.08 ± 2.35	9.59 ± 2.63	9.4 ± 2.6	9.92 ± 2.89	11.1 ± 2.81	12.39 ± 2.93	11.55 ± 2.31	254.87	<0.001
MSCEIT	9.54 ± 3.34	9.54 ± 3.2	9.36 ± 3.06	9.56 ± 2.85	9.75 ± 2.84	11.2 ± 2.54	11.27 ± 2.69	9.98 ± 3.27	76.74	<0.001

ANCOVA, age as covariance;Kruskal-Wallis test.

Among the nine cognitive subtests, the CPT-IP, Category fluency, and MSCEIT Managing Emotions tests achieved their highest scores at 10 years of education. The WMS-III Spatial Span test peaked at 9 years of education, while the remaining subtests showed progressively higher scores with increasing educational attainment. Significant differences were observed across all eight educational levels for all nine cognitive subtests.

### Comparisons of age and gender impact on cognitive performance

A series of multiple linear regression analyses was performed in order to evaluate the influence of age and education attainment on cognitive performance; they are summarized in **Tables 4**. The variables were entered in hierarchical order into a linear regression model using the enter method in order to evaluate the changes in explained variance in all tests.

**Table 4:** Multiple linear regression analysis: comparisons of age and gender impact on cognitive performance.

Test	Variables	β	p	ΔR^2^	Sig. F Change	*Adjusted R* ^2^
TMT.A						
1	gender	-0.155	<0.001	0.024	<0.001	0.023
2	gender	-0.164	<0.001	0.136	<0.001	0.158
	age	0.368	<0.001			
3	gender	-0.168	<0.001	0.009	<0.001	0.166
	age	0.089	0.272			
	education	0.295	<0.001			
BACS.SC						
1	gender	0.054	0.056	0.003	0.056	0.002
2	gender	0.04	0.086	0.325	<0.001	0.327
	age	0.57	<0.001			
3	gender	0.034	0.14	0.021	<0.001	0.347
	age	0.144	0.045			
	education	0.45	<0.001			
HVLT						
1	gender	-0.014	0.622	0	0.622	-0.001
2	gender	-0.022	0.412	0.104	<0.001	0.102
	age	0.322	<0.001			
3	gender	-0.027	0.314	0.013	<0.001	0.115
	age	-0.017	0.841			
	education	0.357	<0.001			
WMS.III.SS						
1	gender	-0.112	<0.001	0.013	<0.001	0.012
2	gender	-0.118	<0.001	0.054	<0.001	0.065
	age	0.232	<0.001			
3	gender	-0.121	<0.001	0.004	0.021	0.068
	age	0.044	0.61			
	education	0.198	0.021			
NAB.Mazes						
1	gender	-0.243	<0.001	0.059	<0.001	0.058
2	gender	-0.25	<0.001	0.089	<0.001	0.146
	age	0.298	<0.001			
3	gender	-0.254	<0.001	0.007	0.002	0.152
	age	0.056	0.493			
	education	0.255	0.002			
BVMT						
1	gender	0.047	0.096	0.002	0.096	0.001
2	gender	0.042	0.128	0.037	<0.001	0.038
	age	0.192	<0.001			
3	gender	0.041	0.142	0.001	0.19	0.038
	age	0.084	0.334			
	education	0.114	0.19			
Fluency						
1	gender	-0.004	0.885	0	0.885	-0.001
2	gender	-0.014	0.584	0.164	<0.001	0.162
	age	0.405	<0.001			
3	gender	-0.019	0.451	0.015	<0.001	0.177
	age	0.041	0.607			
	education	0.383	<0.001			
MSCEIT.ME						
1	gender	0.014	0.613	0	0.613	-0.001
2	gender	0.01	0.728	0.034	<0.001	0.033
	age	0.185	<0.001			
3	gender	0.007	0.79	0.003	0.055	0.035
	age	0.027	0.755			
	education	0.167	0.055			
CPT						
1	gender	-0.06	0.034	0.004	0.034	0.003
2	gender	-0.075	0.001	0.342	<0.001	0.344
	age	0.585	<0.001			
3	gender	-0.081	<0.001	0.023	<0.001	0.367
	age	0.131	0.064			
	education	0.479	<0.001			

Associations of gender, age and education attainments with cognitive performance. Linear regression model. Variables entered in a hierarchical way in 3 blocks—1. gender, 2. age, 3. education attainments; The variable p, the β, adjusted R^2^ and ΔR^2^ are the values after each step.

After adjusting for gender, age emerged as a significant predictor of performance across all tests (β range = 0.185 to 0.585; all p < 0.05). However, after further adjustment for educational attainment, the effect of age was no longer significant in 8 out of the 9 tests (β range = 0.017 to 0.131; all p > 0.05), with the exception of the BACS.SC (β = 0.144; all p =0.045).

Age accounted for 3.3% to 34.4% of the variance in cognitive performance across all tests, with each model exhibiting a significant increment in ΔR² (all p < 0.001). Following additional adjustment for educational attainment, the models corresponding to 7 out of 9 tests explained 6.8% to 36.7% of the variance, accompanied by a significant ΔR² increment (all p < 0.001), the only exceptions were the models for BVMT and MSCEIT.ME.

## Discussion

This study establishes the first regionally representative normative standards for the MATRICS Consensus Cognitive Battery (MCCB) among Chinese adolescents aged 11–16 years. Our results document three fundamental patterns of cognitive development: significant age-dependent trajectories, domain-specific gender differences, and education-modulated performance profiles. These findings provide critical insights for clinical assessment protocols and neurodevelopmental research.

Our study demonstrates age-related cognitive improvements across most MCCB domains in Chinese adolescents. Although we found significant overall differences between age groups, *post hoc* tests showed no significant pairwise differences, indicating gradual developmental changes. This continuous progression pattern contrasts with some previous studies reporting more substantial cognitive gains during adolescence (25), a discrepancy that may stem from methodological differences in age grouping, where prior work used 4-year intervals versus our 1-year stratification (25), allowing for more sensitive detection of incremental changes. Notably, our findings of progressive but decelerating cognitive maturation align with established meta-analytic evidence demonstrating a characteristic developmental sequence: rapid cognitive improvements during childhood transition to more moderated gains in adolescence, ultimately reaching a relative plateau in late adolescence and young adulthood (26). This nonlinear trajectory reflects well-documented neurodevelopmental processes, particularly the extended maturation timeline of prefrontal circuits that underlie executive functions (27). The observed pattern corresponds to initial robust neural reorganization during early adolescence, marked by intensive myelination and synaptic pruning, followed by more gradual refinement continuing through late adolescence (27, 28).

A noteworthy exception was the TMT-A, which exhibited no age effect. This is likely attributable to ceiling effects in older adolescents, as TMT-A primarily assesses visual tracking and basic motor coordination—abilities that may mature relatively early in adolescence. At this stage, most adolescents have already reached near-optimal performance on such tasks, limiting the test’s sensitivity to further age-related improvements.

Males outperformed females in psychomotor speed (TMT-A), sustained attention (CPT-IP) and spatial tasks (WMS-III SS, NAB Mazes) while females excelled in visuospatial memory (BVMT-R). Regarding males’ superior performance in NAB Mazes, our findings are consistent with those from studies on American adolescent norms and Chinese adult norms (5, 21). These findings mirror meta-analytic results (29), there is ample evidence of the influence of sex hormones on visual-spatial working memory in humans. Males demonstrated faster information processing speed, which was inconsistent with the performance observed in Norwegian norms, yet this discrepancy was not detected in studies conducted in the United States (20, 21). Additionally, no significant gender differences were identified in social cognition (MSCEIT), which might be attributed to the high homogeneity in improvements related to social cognitive abilities, which weakened the manifestation of potential gender differences.

Notable disparities in cognitive abilities were observed across levels of educational attainment. After adjusting for age, significant differences persisted in all subtests. Despite the uneven distribution of sample sizes across educational attainments due to age-based participant selection, the study still had an adequate sample size in each subgroup categorized by educational level. We found that all test performances, including the TMT-A, exhibited significant differences among the eight groups. In future comparative studies on the TMT-A, researchers are advised to analyze variations in test performance based on educational attainment rather than age, thereby enhancing the relevance and explanatory power of the results. Improvements in cognitive abilities associated with educational attainment may stem from the pivotal role of school quality and curricular depth in the cognitive development of Chinese adolescents.

After adjusting for gender, age initially exhibited significant associations with all cognitive tests; however, upon further adjustment for education, the effect of age was no longer significant in eight of the nine tests. Our findings highlight the predominant role of educational attainment over age in predicting cognitive performance. This pattern suggests that education largely cover the relationship between age and cognitive function. Moreover, the incremental variance explained by education was statistically significant in seven tests, reinforcing its stronger independent influence. These results underscore the importance of educational attainment as a key factor explain age-related cognitive differences.

The establishment of age-specific normative data enables sensitive detection of subtle cognitive impairments in adolescents at clinical risk for psychosis, particularly due to cognitive deficits that frequently emerge before overt symptom manifestation (30, 31). These robust reference standards enhance the MCCB’s clinical utility for both diagnostic assessment and intervention monitoring in adolescent populations. Findings of this study indicate that cognitive function in most domains improves continuously with age. A “high-risk” signal may also include the absence of age-expected progress in cognitive function. By acquiring data on MCCB performance in healthy adolescents, it is possible to elucidate the differential trajectories of cognitive function in early-stage psychosis, thereby facilitating the formulation of individualized cognitive rehabilitation interventions (32). Our fine-grained age stratification (1-year intervals) offers unprecedented sensitivity for tracking neurodevelopmental trajectories during this critical period, delivering clinically meaningful prognostic insights for early-onset schizophrenia cases.

The study sample was exclusively drawn from the urban area of Xiaogan, and did not include any rural populations. This limits the applicability of the local urban norm to rural areas of Xiaogan. Although the demographic and educational characteristics of Xiaogan are closely aligned with those of Hubei Province as a whole, the applicability of the norms to rural area of Xiaogan must be established through further validation. Consequently, the normative data are specific to urban Xiaogan and not generalizable to its rural areas. Furthermore, the clinical utility of these norms requires additional investigation through longitudinal studies examining their predictive validity for identifying psychosis conversion among high-risk adolescent populations.

In conclusion, by providing robust developmental norms for MCCB performance in Chinese adolescents, this study fills a critical gap in neurocognitive assessment tools. Our data underscore the interplay of age, gender, and education in shaping cognitive trajectories, offering a foundation for early identification of neurodevelopmental disorders and cross-cultural research on adolescent brain maturation.

## Data Availability

The original contributions presented in the study are included in the article/supplementary material. Further inquiries can be directed to the corresponding author.
